# Transcriptome-Wide Survey and Expression Profile Analysis of Putative Chrysanthemum HD-Zip I and II Genes

**DOI:** 10.3390/genes7050019

**Published:** 2016-05-17

**Authors:** Aiping Song, Peiling Li, Jingjing Xin, Sumei Chen, Kunkun Zhao, Dan Wu, Qingqing Fan, Tianwei Gao, Fadi Chen, Zhiyong Guan

**Affiliations:** College of Horticulture, Nanjing Agricultural University, Nanjing 210095, China; aiping_song@aliyun.com (A.S.); lxmlpl@163.com (P.L.); 2014104098@njau.edu.cn (J.X.); chensm@njau.edu.cn (S.C.); 2014104099@njau.edu.cn (K.Z.); 2013104120@njau.edu.cn (D.W.); 2013104100@njau.edu.cn (Q.F.); 2014104101@njau.edu.cn (T.G.); chenfd@njau.edu.cn (F.C.)

**Keywords:** *Chrysanthemum morifolium*, HD-Zip, phylogenetic analysis, stress response

## Abstract

The homeodomain-leucine zipper (HD-Zip) transcription factor family is a key transcription factor family and unique to the plant kingdom. It consists of a homeodomain and a leucine zipper that serve in combination as a dimerization motif. The family can be classified into four subfamilies, and these subfamilies participate in the development of hormones and mediation of hormone action and are involved in plant responses to environmental conditions. However, limited information on this gene family is available for the important chrysanthemum ornamental species (*Chrysanthemum morifolium*). Here, we characterized 17 chrysanthemum *HD-Zip* genes based on transcriptome sequences. Phylogenetic analyses revealed that 17 *CmHB* genes were distributed in the HD-Zip subfamilies I and II and identified two pairs of putative orthologous proteins in *Arabidopsis* and chrysanthemum and four pairs of paralogous proteins in chrysanthemum. The software MEME was used to identify 7 putative motifs with E values less than 1e-3 in the chrysanthemum HD-Zip factors, and they can be clearly classified into two groups based on the composition of the motifs. A bioinformatics analysis predicted that 8 *CmHB* genes could be targeted by 10 miRNA families, and the expression of these 17 genes in response to phytohormone treatments and abiotic stresses was characterized. The results presented here will promote research on the various functions of the *HD-Zip* gene family members in plant hormones and stress responses.

## 1. Introduction

Transcription factors (TFs) play crucial roles in plant development, growth, and responses to various environmental conditions. The homeodomain-leucine zipper (HD-Zip) transcription factor family is one of the key transcription factor families [1], and it is unique to the plant kingdom, although it is found in the recently identified charophycean algae [2]. Members of the HD-Zip family contain a special conserved HD domain that is responsible for its specific binding to DNA and an adjacent leucine zipper motif (LZ) that is a dimerization motif. The homeodomain is a 60–61 amino acid DNA-binding domain composed of three alpha helices, wherein the second and third helices form a helix-turn-helix DNA-binding motif capable of interacting specifically with DNA [3]. The HD-Zip proteins bind to DNA as dimers, and the LZ motif promotes the formation of homo- and hetero-dimers for the efficient recognition of DNA.

The HD-Zip family can be classified into four subfamilies, HD-Zip I to IV, based on four distinguishing characteristics: HD-Zip domain conservation, additional conserved domains, gene structures and physiological functions [4]. The HD-Zip I proteins exhibit a highly conserved HD, a less conserved LZ and no other similarities. The HD-Zip II subfamily contains two additional motifs: the CPSCE (named after the five conserved amino acids Cys, Pro, Ser, Cys, Glu in the one letter code) and an N-terminal consensus sequence. Compared with subfamily I, subfamily III shows additional amino acids in the binding domain between the HD and the LZ motifs; this binding domain includes a conserved START (steroidogenic acute regulatory protein-related lipid transfer) domain, an adjacent conserved SAD (START-adjacent domain) region, and a MEKHLA domain in the C-terminus. Subfamily IV proteins are HD-Zip-START-SAD and similar to those of subfamily III, although the most distinguishable features are the presence of a loop in the middle of the LZ domain and the lack of an additional C-terminal MEKHLA motif. The proteins encoded by HD-Zip I form dimers that recognize the pseudo palindromic sequence CAAT(A/T)ATTG [5]. The HD-Zip II proteins bind to the same sequence and differ from the members of the HD-Zip I family only in the recognized nucleotide located in the center of the target site [6]. HD-Zip III proteins can interact with GTAAT(G/C)ATTAC *in vitro* [7], whereas HD-Zip IV proteins show a binding preference for the alternative sequence TAAATG(C/T)A [8].

Many members of the HD-Zip protein have been analyzed in various plant species, including *Arabidopsis* (*Arabidopsis thaliana*) [9], rice (*Oryza sativa*) [10], maize (*Zea mays*) [11], tobacco (*Nicotiana sylvestris*) [12], tomato (*Solanum lycopersicum*) [13], barley (*Hordeum vulgare*) [14], soybean (*Glycine max*) [15], *Medicago truncatula* [16], sunflower (*Heliantus annuus*) [17], moss (*Physcomitrella patens*) [18], and poplar (*Populus trichocarpa*) [19]. The structural and functional characterization of HD-Zip proteins was limited to *Arabidopsis* when only a few HD-Zip proteins had been identified in other species. Currently, there are 47 HD-Zip transcription factors known in *A. thaliana*, including 17 members of HD-Zip I, 9 members of HD-Zip II, 5 members of HD-Zip III, and 16 members of HD-Zip IV, and they play various roles in plant development.

The expression of HD-Zip I proteins is regulated by abiotic stresses, including drought, extreme temperatures, osmotic stress and illumination conditions, and the proteins are involved in responses to these environmental conditions as well as de-etiolation [20]. For example, *ATHB1*, the first member of HD-Zip, functions as a mediator in the determination of leaf cell fate [21]. Zhao *et al*. found 17 HD-Zip transcription factors in maize that were regulated by drought stress [11]. A novel maize HD-Zip I gene, *Zmhdz10*, which is induced by drought, salt stress and abscisic acid (ABA), can positively regulate drought and salt tolerance in plants through an ABA-dependent signaling pathway [22]. *ATHB5* might contribute to the spatial regulation of *BDL* expression to modulate the BDL-dependent auxin response [23]. An *in situ* hybridization analysis of *Vrs1* from barley indicated that it encodes an HD-Zip I transcription factor and is expressed exclusively in the pistil, lemma, palea and lodicule of the lateral spikelets; moreover, *Vrs1* was also found to inhibit gynoecial development, thus showing the neofunctionalization of the HD-Zip I subfamily [24].

HD-Zip II proteins are mainly involved in plant responses to illumination conditions, shade avoidance and auxin signaling [25]. *ATHB2* can be induced rapidly by changes in the red to far-red ratio to promote the shade-avoidance response in the majority of angiosperms [9], whereas *HAT2* is involved in the regulation of auxin-mediated morphogenesis in the shoot and root tissues [26]. Furthermore, recent studies have revealed additional functions of this subfamily in plant development, including carpel margin development [27] and leaf polarity [28]. The progressive loss of activity of HOMEOBOX 2 (ATHB2), HAT3 and ATHB4 (HD-Zip II proteins in *Arabidopsis*) in *A. thaliana* causes developmental defects during embryogenesis, suggesting that these regulators control apical embryo development and meristem regulation [29].

HD-Zip III proteins are regulators of apical meristem, embryogenesis, auxin transport, leaf polarity, later organ initiation and vascular system development; thus, they play overlapping, antagonistic or distinct roles [30,31]. Plants that overexpress *ATHB8* show an overproduction of xylem, indicating that this gene plays a crucial role in vascular development [32]. *PtaHB1* overexpression produces noticeable effects on petiole and primary shoot fiber development, suggesting that *PtaHB1* is involved in secondary vascular growth in angiosperms and gymnosperms [31]. The *popREVOLUTA* (*PRE*) gene from *Populus* plays a fundamental role in the initiation of the cambium and the regulation of secondary vascular tissue patterning [33].

HD-Zip IV proteins play crucial roles in epidermal cell differentiation, anthocyanin accumulation, root development, trichome formation and cuticle development [34]. The HD-Zip IV gene *HDG11* can improve drought tolerance and increase grain yield in transgenic rice plants [35]. *ATML1* regulates gene expression in the epidermis specification [8], *PROTODERMAL FACTOR2* (*PDF2*) is crucial for normal development of floral organs in *Arabidopsis* [36]. Moreover, *ATML1* and *PDF2* act redundantly as a positive regulator of shoot epidermal cell differentiation and at least one copy of these genes is essential for embryo development [37]. *GLABRA2* (*GL2*) is required for the differentiation of epidermal cells in *Arabidopsis* tricomes, and it activates a positive feedback loop via MYB23 [38].

*HD-Zip* genes are also involved in regulating the adaptive response of plants to biotic stresses, including the microbes *Pseudomonas syringae* [39] and *Alternaria alternate* [40] and the insects *Spodoptera littoralis* and *S. frugiperda* [41].

Recently, a genome-wide and expression analysis of the HD-Zip transcription factors was performed for various species, including peach with 33 members [42], pear with 52 members [40] and soybean with 101 members [43]. The RNA-seq approach indicated that five *HD-Zip I* genes and one *HD-Zip II* gene showed differential expression under dehydration stress, while seven *HD-Zip I*, four *HD-Zip II*, one*HD-Zip III*, and four *HD-Zip IV* genes showed differential expression under salt stress in soybean [43]. However, the systematic identification of sequences and expression patterns under abiotic stress has not been conducted for chrysanthemum. Chrysanthemum (*Chrysanthemum morifolium*) is one of the four most famous cut flowers in the world, and a perennial Asteraceae species; however, it is susceptible to various biotic and abiotic stresses [44]. Asteraceae is one of the largest families of flowering plants with more than 23,000 species, but rare information was available about *HD-Zip* gene family. For this study, we isolated 17 *HD-Zip* genes in chrysanthemum based on a set of transcriptome data. We performed a comparative phylogenetic analysis of chrysanthemum and *Arabidopsis* genes *in silico* and investigated the transcript levels in response to various phytohormones and abiotic stresses using qRT-PCR. The results provided novel insights into the stress responses of *CmHB* genes and provided a better understanding of the structure and function of the HD-Zip factors in chrysanthemum plants.

## 2. Materials and Methods

### 2.1. Plant Materials and Growth Conditions

Cuttings of the cut-flower chrysanthemum cultivar “Jinba”, which is maintained at the Chrysanthemum Germplasm Resource Preservation Center (Nanjing Agricultural University, Nanjing, China), were rooted in vermiculite plus plain water without fertilizer in a greenhouse. After 14 days, the plants were transplanted to a growth substrate (1:1 mixture of garden soil and vermiculite) and subjected to a range of stress and phytohormone treatments.

### 2.2. Plant Treatments

Tissue-specific and treatment-induced transcription profiles of 17 *CmHB* genes were explored in the roots, stems and leaves of young seedlings as well as in the tube and ray florets of inflorescences at the bud stage. A variety of abiotic stresses were imposed, including high salinity (200 mM NaCl) and drought (20% w/v polyethylene glycol (PEG6000)) [45].

For the NaCl and PEG6000 stresses, the six-to-eight-leaf stage plants were transferred to liquid medium containing the stress agent, and the second true leaves were sampled at various times [46]. The wounding treatment involved cutting the second true leaf, and the phytohormone treatments involved spraying the leaves with 50 μM ABA, 1 mM methyl jasmonate (MeJA) or 200 μM salicylic acid (SA) [47]. The plants were sampled prior to the treatment and then at 1, 4, 12 and 24 h after the treatment.

After sampling, all of the collected material was snap frozen in liquid nitrogen and stored at −70 °C. Each treatment was replicated three times.

### 2.3. Transcriptome Search and Sequencing of Full-Length *CmHB* cDNAs

All of the putative HD-Zip proteins were retrieved from *C. morifolium* transcriptome data [48]. *Arabidopsis* HD-Zip protein sequences were downloaded from The *Arabidopsis* Information Resource (TAIR) database. The chrysanthemum transcriptome was searched to identify HD-Zip proteins using Basic Local Alignment Search Tool algorithms (tBLASTx) and the published *Arabidopsis* HD-Zip protein sequences as query sequences. All of the obtained protein sequences were examined for the presence of the HD and LZ domains using the Pfam (http://pfam.sanger.ac.uk/search) and SMART (http://smart.embl-heidelberg.de/) tools. Multiple alignments among the identified *CmHB* sequences were also performed to avoid repetition. Furthermore, the full open reading frames of the *CmHB* sequences were obtained via RACE PCR. First-strand cDNA was synthesized using the dT adaptor primer dT-AP and then subjected to nested PCR using the primer pair CmHBx-3-F1/F2 and the adaptor primer AP (Table S1). Finally, 17 pairs of gene-specific primers (Table S2) were designed to amplify complete open reading frames. The amplicons were purified using AxyPrep DNA Gel Extraction Kits (Axygen, Hangzhou, China) and cloned into pMD19-T (TaKaRa, Tokyo, Japan) for sequencing.

### 2.4. Phylogenetic Tree Construction and Sequence Analysis

A phylogenetic tree was constructed with MEGA version 6.0 using the maximum likelihood method [49]. ClustalW software was employed for multi-sequence alignments of HD-Zip I & II TFs between *Arabidopsis* and *C. morifolium* [50]. Internal branching support was estimated using 500 bootstrap replicates. The theoretical isoelectric point (pI) and molecular weight (Mw) of the CmHB proteins were calculated using the Compute pI/Mw online tool (http://web.expasy.org/compute_pi/), and subcellular localization was predicted with PSORT [51]. Putative conserved motifs were predicted using the MEME program v4.10.2 [52] with the following parameters: any number of repetitions; motif sites, at least 3 sites; optimum motif widths between 6 and 61 residues; and E-value less than 1e-3. All of the motifs identified by MEME were searched in the InterPro database using InterProScan [53]. The target prediction for miRNA was performed using the psRNATarget online tool [54].

### 2.5. Real-Time Quantitative PCR (qPCR)

Total RNA was isolated from the samples using the RNAiso reagent (TaKaRa) according to the manufacturer’s instructions. The RNA was then treated with RNase-free DNase I (TaKaRa) to remove potential genomic DNA contamination. First-strand cDNA was synthesized from 1 μg of total RNA using SuperScript III reverse transcriptase (Invitrogen, Carlsbad, CA, USA) according to the manufacturer’s instructions. The qPCR was performed using a Mastercycler ep realplex instrument (Eppendorf, Hamburg, Germany). Each 20 μL amplification reaction contained 10 μL of SYBR^®^ Premix Ex Taq^™^ II (TaKaRa), 0.4 μL of each primer (10 μM), 4.2 μL of H_2_O and 5 μL of cDNA template. The PCR cycling regime consisted of an initial denaturation (95 °C for 2 min) followed by 40 cycles of 95 °C for 10 s, 55 °C for 15 s and 72 °C for 20 s. A melting curve analysis was performed following each assay to confirm the specificity of and efficiency of the primer pairs. Gene-specific primers (provided in Table S3) were designed using Primer3 Release 2.3.4 [55], and the *EF1α* gene was employed as a reference sequence [47]. The relative transcript abundance was calculated by the 2^−ΔΔ*C*T^ method [56]. Three independent experiments were performed.

### 2.6. Data Analysis

The relative expression levels of each *CmHB* gene were log_2_ transformed. The profiles were compared using Cluster v3.0 software [57] and visualized using Treeview [58]. SPSS v17.0 software (SPSS Inc., Chicago, IL, USA) was utilized for all statistical analyses.

## 3. Results

### 3.1. Identification and Phylogenetic Analysis of Putative HD-Zip Factors in Chrysanthemum

Seventeen chrysanthemum *HD-Zip* gene sequences were isolated and designated *CmHB1* through *CmHB17* (GenBank: KT253049–KT253065). The full-length cDNAs varied in length from 708 to 1263 bp, and the predicted protein products ranged from 166 (CmHB11) to 337 (CmHB17) amino acids. Details regarding the *CmHB* sequences are given in Table 1. All of the CmHB proteins are predicted to be localized in the nucleus.

**Figure 1:**
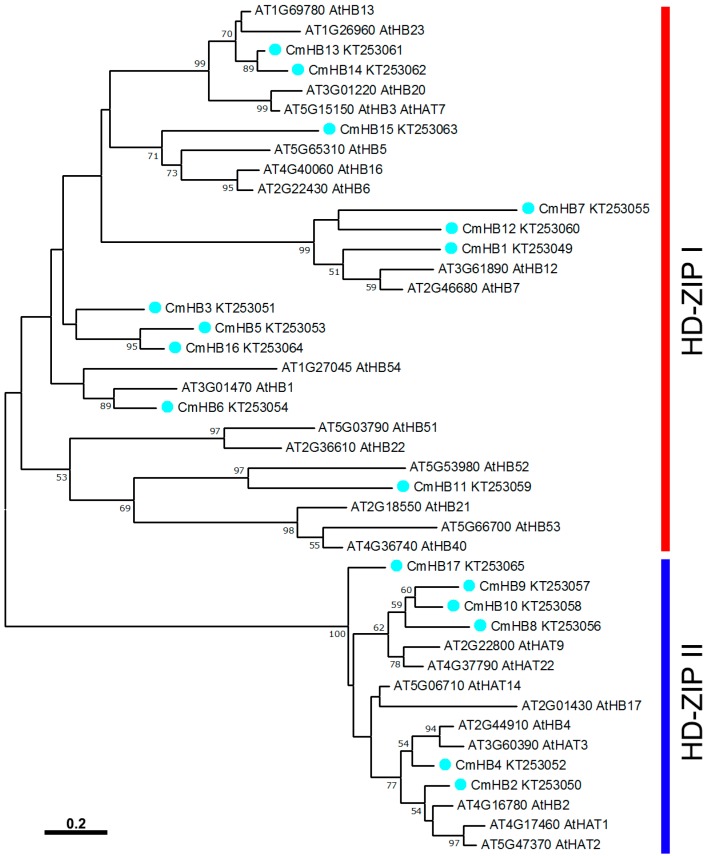
Phylogenetic tree for the Arabidopsis and chrysanthemum HD-Zip I & II proteins. The tree was constructed from a complete alignment of 26 *Arabidopsis* and 17 chrysanthemum HD-Zip proteins using the Maximum Likelihood method. Bootstrap values below 50% have been omitted.

To evaluate the evolutionary relationships between the *Arabidopsis* and chrysanthemum HD-Zip I and II subfamily proteins, the deduced amino acid sequences of the identified *HD-Zip* genes were completely aligned. A combined phylogenetic tree (Figure 1) was then constructed using the Maximum Likelihood method and bootstrap analysis (500 reiterations), and the results showed diversification within the plant family (Figure 1). The seventeen *CmHB* genes were found to be distributed in the HD-Zip subfamilies I and II. Furthermore, two pairs of orthologous proteins were identified in *Arabidopsis* and chrysanthemum (AtHB1 with CmHB6 and AtHB52 with CmHB11), and four pairs of paralogous HD-Zip family proteins were identified in chrysanthemum (CmHB7 with CmHB12, CmHB9 with CmHB10, CmHB13 with CmHB14 and CmHB5 with CmHB16).

### 3.2. Conserved Sequences in Chrysanthemum HD-Zip Proteins

**Figure 2:**
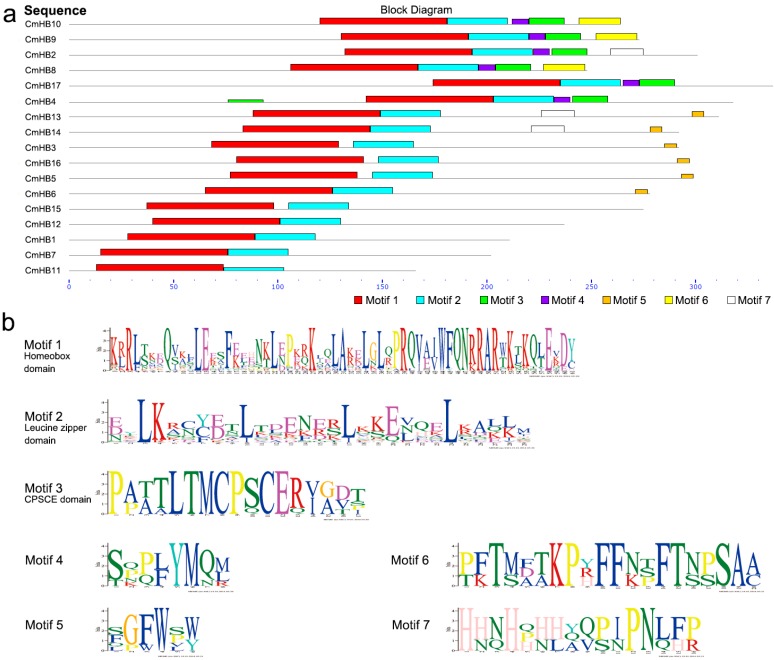
Chrysanthemum HD-Zip protein motifs derived from the MEME analysis. (**a**) Distribution of conserved motifs in HD-Zip proteins; (**b**) sequences of motifs of HD-Zip proteins.

The software MEME was used to predict the motif composition of the HD-Zip factors, and 7 putative motifs with E values less than 1e-3 were identified (Figure 2). The HD-Zip TFs of the chrysanthemum can be clearly classified into two groups based on the motif composition (Figure 2a). For example, Motif 5 is only shared among the HD-Zip I subfamily members, whereas Motifs 3, 4, and 6 are shared among the HD-Zip II subfamily members. All of the chrysanthemum HD-Zip proteins have Motif 1 and Motif 2. Details of these motif features are shown in Figure 2b. The conserved sequences of these motifs were searched against the InterPro database. Motif 1 matched a homeobox domain, Motif 2 matched a leucine zipper domain, and Motif 3 matched a CPSCE domain; however, significant matches were not retrieved for the other motifs.

### 3.3. miRNA Target Site Prediction

All of the available plant miRNA data were used to predict candidates targeting *CmHB* transcripts. As shown in Table 2, 8 *CmHB*s are predicted to be targeted by 10 miRNA families. *CmHB9* contains three target sites, *CmHB4* contains two target sites, and the remaining 6 *CmHB*s contain only one target site. Additionally, miR414 can target *CmHB4* and *CmHB9*.

### 3.4. Transcription Profiling of *CmHB* Genes

**Figure 3:**
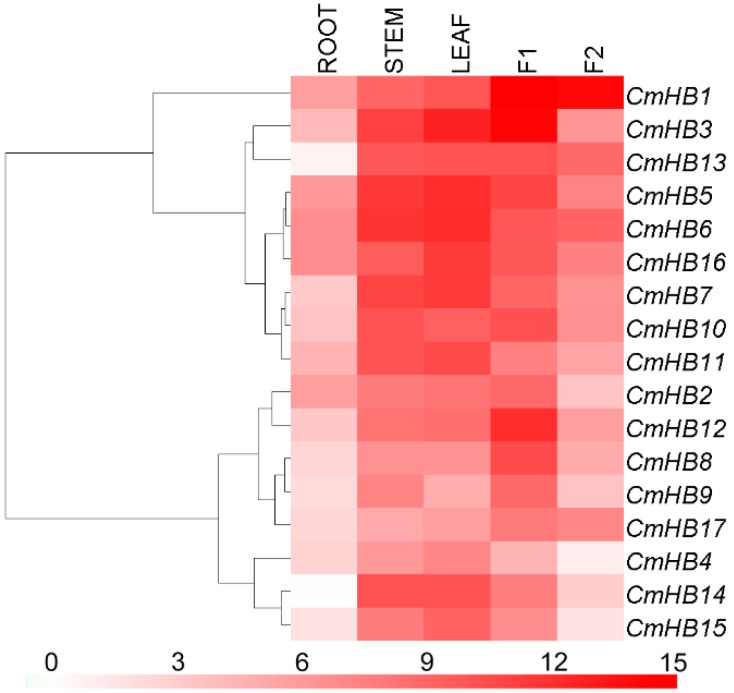
Differential transcription of *CmHB* genes. F1, tubular florets; F2, ray florets at the budding stage. White and red indicate lower and higher transcript abundance, respectively. The relative expression levels of each *CmHB* gene were log2 transformed, the lowest expression level was defined as control (zero).

Because HD-Zip factors in chrysanthemum have not been documented previously, we investigated the expression profiles of these genes. The results showed differential expression of the 17 *CmHB* genes throughout the plant (Figure 3). However, the expression of *CmHB1* in the tubular florets was more than three orders of magnitude higher than that of *CmHB14* in the roots. Interestingly, the *CmHB5* and *CmHB16* paralogs exhibited a similar expression pattern, whereas the *CmHB13* and *CmHB14* paralogs showed different patterns.

### 3.5. Expression of *CmHB* Genes After Treatment with Phytohormones

**Figure 4:**
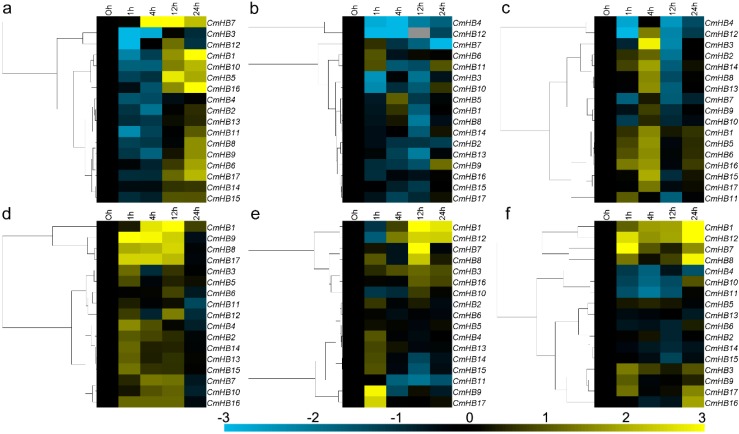
Differential transcription of *CmHB* genes in leaves induced by the exogenous supply of (**a**) abscisic acid (ABA), (**b**) methyl jasmonate (MeJA), (**c**) salicylic acid (SA), (**d**) salinity stress, (**e**) osmotic stress, and (**f**) wound treatments. Blue and yellow indicate lower and higher transcript abundance compared with the relevant controls, respectively. Grey blocks indicate that the transcription was not detected.

*CmHB7* was significantly induced after 4 h of ABA treatment, although *CmHB3, 11,* and *12* were repressed at 1 h. The *CmHB2* genes were down-regulated by exogenous ABA at 4 h, although *CmHB6* and *CmHB17* were induced at 24 h. In addition, the *CmHB1, 5, 8, 9, 10,* and *16* transcripts decreased at 1 h and/or 4 h but increased at 12 h and/or 24 h after ABA treatment, whereas *CmHB4*, *13*, *14*, and *15* expression was not affected by ABA (Figure 4a). *CmHB3*, *4*, *7*, *10* and *12* were strongly down-regulated by MeJA treatment, whereas *CmHB8*, *13* and *14* were only slightly down-regulated. Although the expression of the other *CmHB*s (1, 2, 6, 11, 15, 16 and 17) was not significantly altered by MeJA treatment, *CmHB5* and *CmHB9* were induced at 4 h and 14 h, respectively (Figure 4b). After SA treatment, *CmHB4* and *CmHB12* were repressed at 1 h and 12 h, whereas *CmHB11* was repressed only at 12 h; however, *CmHB7*, *9* and *12* were not significantly altered. Moreover, the expression levels of the other eleven *CmHB*s increased at 4 h after SA treatment, whereas the levels of *CmHB2*, *3*, *8*, *13* and *14* decreased at 12 h (Figure 4c).

### 3.6. Expression Profiling of *CmHB* Genes Under Abiotic Stress

Three main expression patterns of the *CmHB* genes were observed under salinity stress. Although the expression of *CmHB3, 5, 6,* and *11* was not significantly altered by the NaCl treatment, *CmHB1, 8, 9* and *17* were strongly induced at 24 h, whereas the other nine *CmHB* genes were slightly up-regulated (Figure 4d). Eight *CmHB* genes (*2, 3, 4, 5, 6, 10, 13,* and *16*) were weakly regulated by drought stress and presented a less than 2-fold range of variation. *CmHB9* and *CmHB17* were markedly induced at 1 h after PEG treatment, whereas four *CmHB* genes (*1, 7, 8* and *12*) were induced at 12 h. Furthermore, *CmHB11, 14* and *15* were down-regulated by high osmotic pressure at 12 h (Figure 4e). Three *CmHB* genes (*4*, *10,* and *11*) were significantly repressed by mechanical damage, whereas the transcription of four *CmHB* genes (*1*, *7, 8,* and *12*) was significantly increased. In addition, *CmHB3, 9, 16* and *17* were slightly up-regulated by mechanical damage, whereas the expression of the other 6 *CmHBs* was not significantly altered (Figure 4f).

## 4. Discussion

The HD-Zip gene family has been isolated and characterized in certain plant species, including *Arabidopsis* [3], poplar [19], soybean [15], and rice [10]. However, this family has not previously been studied in chrysanthemum. In the current study, we performed an overall analysis of the *HD-Zip* gene family in the chrysanthemum transcriptome, including an analysis of their phylogeny, conserved motifs and expression profiles. The comparative analysis of the HD-Zip family in *Arabidopsis* and chrysanthemum allowed for the prediction of various functions of the chrysanthemum HD-Zip family members and helped to facilitate further gene function analysis.

### 4.1. Comparative Analysis of the Chrysanthemum and Arabidopsis HD-Zip Gene Families

In this study, a total of 17 full-length HD-Zip genes were identified in chrysanthemum based on transcriptome data. A comparative analysis of the chrysanthemum and *Arabidopsis* *HD-Zip* genes found that seventeen *CmHB* genes distributed in the HD-Zip subfamilies I and II, whereas none were distributed in subfamilies III and IV (Figure 1). This observation may have been caused by limitations of the transcriptome data, which means the expression of III and IV subfamily gene was too low to be detected. All the CmHBs were classified by the presence of a highly conserved homeodomain (Figure 2). In addition, the assessment of the subcellular localization of chrysanthemum HD-Zip proteins revealed strong support for their functional roles in in the regulation of transcription (Table 1). Nonetheless, the transcriptional activity of these family members required additional investigation.

We further analyzed the conserved motifs among the chrysanthemum HD-Zip family members using the MEME program and found that the majority of CmHBs within the same group shared similar motifs (Figure 2), suggesting that these conserved motifs play crucial roles in group-specific functions. However, a high divergence in structure was found among the different groups. As the motif analysis in pear HD-Zip gene family, subfamily III contains the most motifs, whereas subfamilies I and II contain the fewest motifs [40]. Only 7 putative motifs with E values less than 1e-3 were identified in subfamilies I and II, and the position of the motifs among the members of an individual subfamily was conserved. These two features of chrysanthemum are consistent with those of other species [40].

Previous studies suggested that tandem and segmental duplications play a substantial role in the expansion of gene families during the process of genome evolution [11]. Each chrysanthemum HD-Zip subfamily presented a unique motif and a similar motif composition, which suggests that the gene family might have expanded by duplication.

### 4.2. miRNA Target Site Prediction

In *Arabidopsis*, HD-Zip III subfamily genes are the targets of two miRNAs, miRNA165 and miRNA166, and this characteristic is conserved in other plants as well [1]. However, reports of miRNA with HD-Zip I and II subfamily interactions are rare. According to our predictions, 8 *CmHB* genes should be targeted by 10 miRNA families (Table 2), which suggests that other interaction modules might occur in the HD-Zip I and II subfamilies. However, the regulation pathways for these interactions in plants should be verified by further research.

### 4.3. Expression Patterns of *CmHB* Genes

Because gene expression patterns can provide important clues for gene functions, we employed qRT-PCR to examine the expression of the *CmHB* genes in the roots, stems and leaves of young seedlings as well as in the tube and ray florets of inflorescences at the bud stage (Figure 3). The expression profiles revealed spatial variations of *CmHB* expression in different organs. Furthermore, a pair of paralogous genes (*CmHB5* and *CmHB16*) exhibited distinct expression patterns, suggesting that significant functional divergence might have occurred following the duplication events [59].

*AtHB1* is highly expressed throughout the pavement, basal and trichome cells of the mature leaves during leaf development [60], and its orthologue in chrysanthemum, *CmHB6*, was also expressed at the highest level in leaves (Figure 3). Furthermore, the other homologs of *AtHB1*, *CmHB3*, *5*, and *16* were highly expressed in the leaves (Figure 3), suggesting they may have functional redundancy in leaf morphogenesis. AtHB4 and HAT3, two class II HD-ZIP transcription factors, also control leaf development in *Arabidopsis*, and their closest homolog *CmHB4* has the highest expression level in the leaves among different organs (Figure 3), which implies that these genes have not yet undergone functional divergence. However, additional research is required to determine the functions of these *CmHB* genes.

The alignment of full-length protein sequences have confirmed that the HD-Zip II proteins can be distributed into two subfamilies, one consisting of HAT22 and HAT9 and the other consisting of HATI, AtHB4, HAT3 and AtHB2 [61]. Expression studies using microarrays have shown that HAT22 expression is up-regulated during drought in *Arabidopsis* [62]. However, there is little functional evidence to suggest a role for HD-Zip II TFs in response to water deficit [63]. The homolog of *Arabidopsis* *HAT22* in *Medicago truncatula*, *MtHB2*, was induced by drought and salt stresses, and it functioned as a negative regulator in plant drought and salt tolerance [16]. The homologs of *MtHB2* in chrysanthemum, *CmHB8* and *CmHB9*, had similar expression patterns and were also induced by drought and salt stress (Figure 4).

ABA is extensively involved in the response to various biotic and abiotic stresses, including pathogen infection, cold, and osmotic stress [64]. *AtHB7* and *AtHB12* are strongly induced by water-deficit and ABA, and they exhibit essential functions as mediators of a negative feedback effect on ABA signaling in the plant response to water deficit [65]. The closest homologs of *AtHB7* in chrysanthemum, *CmHB1* and *CmHB7*, were also significantly induced by ABA and osmotic stress treatments (Figure 4a,e), suggesting that they may have similar functions in the chrysanthemum. However, the homolog of *AtHB12, CmHB12*, was induced by osmotic stress but suppressed by ABA, suggesting that there may be functional divergence between *AtHB12* and *CmHB12*. *AtHB6* is expressed constitutively in the seedlings but significantly up-regulated in the seedlings subjected to water deficit, osmotic stress or exogenous treatment with ABA [66]. However, the expression of *CmHB15* was not affected by ABA, and it was down-regulated by high osmotic pressure at 12 h (Figure 4). This response suggests that *CmHB15* may have a distant phylogenetic relationship with *AtHB6*, and this relationship was also implied by phylogenetic analysis (Figure 1).

SA and MeJA present coordinated functions in biotic stress signaling upon pathogen infection by activating the transcription of several defense-related genes [67]; however, no report has focused on the responses of the *Arabidopsis* *HD-Zip* gene family members to the two hormones. *Helianthus annuus* (sunflower) *HAHB4* is induced by JA, wounding and insect attack, and can negatively regulating SA accumulation [41]. Its homologs in chrysanthemum, *CmHB1* and *CmHB7*, were strongly induced by wounding, but not by JA. Furthermore, its homologs in *Arabidopsis*, *AtHB7 and AtHB12*, mediate a growth response to water deficit [68]. We therefore suggested *CmHB1* and *CmHB7* may experience neofunctionalization, with respect to *HAHB4*. We investigated the responses of *CmHB* genes to SA and MeJA, and the results showed that the *CmHB* genes were up- or down-regulated by the exogenous supply of hormones (Figure 4b,c), thus indicating that *CmHB* genes may be involved in responses to various plant hormones that elicit a stress response.

## 5. Conclusions

To our knowledge, this study is the first transcriptome-wide analysis of the HD-Zip family in chrysanthemum. The changes in expression of 17 *CmHB* genes in response to a range of phytohormones and abiotic stress treatments were characterized. These findings lay the foundation for future research into the function of *CmHB* genes in plant stress responses, which will promote their application in chrysanthemum breeding.

## Figures and Tables

**Table 1:** Summary of CmHB sequences and the most similar *A. thaliana* homologs.

Gene	GenBank Accession No.	Amino Acid Length (aa)	PI	MW	Subcellular Localization	Most Similar *A. thaliana* Homolog	Locus Name
*CmHB1*	KT253049	211	4.98	24656.44	N(9.05)	*ATHB7*	AT2G46680.1
*CmHB2*	KT253050	301	8.82	33945.17	N(9.30)	*ATHB2*	AT4G16780.1
*CmHB3*	KT253051	292	4.85	34011.51	N(7.93)	*ATHB1*	AT3G01470.1
*CmHB4*	KT253052	318	8.7	35070.59	N(9.40)	*HAT3*	AT3G60390.1
*CmHB5*	KT253053	299	4.71	33985.07	N(7.97)	*ATHB1*	AT3G01470.1
*CmHB6*	KT253054	278	4.66	31919.69	N(8.94)	*ATHB1*	AT3G01470.1
*CmHB7*	KT253055	202	6.61	23500.1	N(8.95)	*ATHB7*	AT2G46680.1
*CmHB8*	KT253056	248	8.6	27814.11	N(9.47)	*HAT22*	AT4G37790.1
*CmHB9*	KT253057	273	8.46	30384.22	N(9.32)	*HAT22*	AT4G37790.1
*CmHB10*	KT253058	264	9.13	29681.66	N(9.36)	*HAT9*	AT2G22800.1
*CmHB11*	KT253059	166	8.85	19298.5	N(8.76)	*ATHB52*	AT5G53980.1
*CmHB12*	KT253060	237	5.45	27259.88	N(8.66)	*ATHB12*	AT3G61890.1
*CmHB13*	KT253061	311	5.91	35462.34	N(9.18)	*ATHB13*	AT1G69780.1
*CmHB14*	KT253062	292	5.76	33627.57	N(8.85)	*ATHB13*	AT1G69780.1
*CmHB15*	KT253063	275	4.69	31595.82	N(8.68)	*ATHB6*	AT2G22430.1
*CmHB16*	KT253064	297	4.91	34061.3	N(7.97)	*ATHB1*	AT3G01470.1
*CmHB17*	KT253065	337	7.63	37486.68	N(9.08)	*HAT14*	AT5G06710.1

**Table 2:** The target prediction of miRNA.

miRNA	Target	Exp	UPE	miRNA Start	miRNA End	Target Start	Target End
miR414	*CmHB4*	2.5	20.266	1	21	512	532
miR414	*CmHB9*	2.5	10.125	1	21	460	480
miR917	*CmHB9*	2.5	18.596	1	20	360	379
miR1023a-3p	*CmHB13*	3	13.468	1	20	135	154
miR444	*CmHB16*	2	9.773	1	21	537	557
miR158a-5p	*CmHB5*	3	19.317	1	19	738	757
miR4369	*CmHB17*	3	16.17	1	20	368	387
miR4993	*CmHB4*	3	17.178	1	21	922	942
miR5298	*CmHB10*	3	5.976	1	22	135	156
miR5380	*CmHB9*	3	1.732	1	23	9	31
miR5751	*CmHB14*	3	16.3	1	20	134	153

Exp: Expectation; UPE: allowed maximum energy to unpair the target site.

## Supplementary Materials

The following are available online at www.mdpi.com/2073-4425/7/5/19/s1, Table S1: Primer sequences used to perform 3’- RACE PCR, Table S2: Primer sequences used to amplify the open reading frames of 17 *CmHB* genes, Table S3: Primer sequences used for the transcription analysis of the 17 *CmHB* genes.
