# Supplementary material for: Transcriptome-Wide Survey and Expression Profile Analysis of Putative Chrysanthemum HD-Zip I and II Genes
Source: Genes (Basel). 2016 May 17;7(5):19. doi: 10.3390/genes7050019 (PMC4880839; doi:10.3390/genes7050019)
Supplement: Supplementary file 1 [file genes-07-00019-s001.docx]

Article

Transcriptome-Wide Survey and Expression Profile Analysis of Putative Chrysanthemum HD-Zip I and II Genes

Aiping Song, Peiling Li, Jingjing Xin, Sumei Chen, Kunkun Zhao, Dan Wu, Qingqing Fan, Tianwei Gao, Fadi Chen and Zhiyong Guan *

Supplementary Material

**Table S1.** Primer sequences used to perform 3’- RACE PCR.

| **Primers** | **Sequences** |
| --- | --- |
| HB14-3F1 | GGGCTTCAACCAAGGCAGAT |
| HB14-3F2 | GAAGAACAAGGAACCAACAGAGTC |
| dT-AP | AAGCAGTGGTATCAACGCAGAGTACTTTTTTTTTTTTTTTT |
| AP | AAGCAGTGGTATCAACGCAGAGTAC |

**Table S2.** Primer sequences used to amplify the open reading frames of 17 *CmHB* genes.

| **Gene** | **Forward Primer** | **Reverse Primer** |
| --- | --- | --- |
| *CmHB1* | CCCTTGGGCACATTCCTC | GCTTATTGCGTCCTCTTAAACTAACTAT |
| *CmHB2* | TTCTCACAATCAATCAATACCCAA | GACCAAATCTGTATAGATCCTAAATCA |
| *CmHB3* | GCAATGGAGGGTGGGATG | GCAAATAGTTGGTCTACAATGGG |
| *CmHB4* | CCTTTGTCCCTCTGCCATATC | CCTACATCAAATCCCACTTTTATCC |
| *CmHB5* | GGTGGTGCCATGTCTGCTC | CCACTCATGGATTCAGGGTGT |
| *CmHB6* | TTTTGGATATGGAATCTGGTCAC | CGACAGCCGAAAGCTACTAAAT |
| *CmHB7* | CGCTCATACTCACCAAGTCATCA | AATCTATACTCTATCGTAAATCCCTCAG |
| *CmHB8* | TATACTATTTCACCTTTTGATCCATAAGAC | TTTAACAAGCTGCCGACGAA |
| *CmHB9* | CATTTCGCTAAATTCCCCTTCT | ATTAACAAGCAGCAGATGGATTAG |
| *CmHB10* | TTCCATTTTCCTTTCATCCTCA | TGACAAACCCTTCAGCACTCTG |
| *CmHB11* | CTAAATATGGATTTTTCACAACCCA | CAACCAAACATCTAGTAAGAAAGAAGAA |
| *CmHB12* | GTCAGAAATCTACCACGAGCCC | AATGTACTTCAAACCACCCCAC |
| *CmHB13* | ATGACTTGCACTGGAATGGC | TGAATACAAGGATTATCAAACGCTA |
| *CmHB14* | TTTTAATGGTGAGCCAAGTGATC | TCAAGTAGAAGAAAGCAACAACATTA |
| *CmHB15* | TTTGACAACCCAGTTGAGATGAA | TCCCGAAAGTACCAACAAAGG |
| *CmHB16* | AAGTTTTGCATGGTTGATTGAGC | ATACTTACTCCTGGATTCAAGATGC |
| *CmHB17* | TTAGATGAAACATGGAGTTGGGTT | TCACATCGAACACCAAATCATAGA |

**Table S3.** Primer sequences used for transcription analysis of the 17 *CmHB* genes.

| **Gene** | **Forward Primer** | **Reverse Primer** |
| --- | --- | --- |
| *CmHB1* | CACGTTGGAAGTCGAAGCAGCTAGAA | CGTCCGATACATTGCCCAGTCCTTTC |
| *CmHB2* | GATGTCGCCGCAATTCTATATGCAGATG | CGAGACCGCTAAGTGAGGATGATTATGG |
| *CmHB3* | CCCACACAAGAGTTGGATTCAAATGCT | GGACACCGAAGAATGGCTAAGTCATCT |
| *CmHB4* | AGCGTTGTTGTGATACATTGACAGAGGA | GGCGGAGGATGAGGAAGAGGATGA |
| *CmHB5* | GCTACAACAGGAACAAGTTGCGGATTG | AGATAACCAGTAAGCGGAGTCACGAAGA |
| *CmHB6* | CAGAAGAGGATGACGGCAGTGATGAC | TCGTGATGATGGTGATCCGCAGTTG |
| *CmHB7* | TCAAGAAGACAGTAGAAGGCACGACTCT | GGCAGTTTCCTCCCATACATTGACACT |
| *CmHB8* | GCAAGAGGTTCAACAACTAAGAGCACAA | TCGTGGTGGCGGAATTGGTATCG |
| *CmHB9* | GCGAGACACTAACGGATGAGAACAGAA | TTCACATGACGGACACATGGTTAAGGT |
| *CmHB10* | CCAGAACAGAAGAGCCAGGACAAAGC | TTCGCATGACGGACACATGGTAAGG |
| *CmHB11* | AGAACACAAGATGCTTCAAGATCGGCTT | ACCTTGGAGTCTCTCAACCTCTCTTTCA |
| *CmHB12* | CGAAGCAAGTGGAACAAGACTACTCAAC | GGATCATCCTCGTCCGAACATATAGCC |
| *CmHB13* | CGAAGGATCGAGCAGCAATAGAAGTGAA | TTGATGGTGTTGGTGTTGATGGTGTGA |
| *CmHB14* | GGCATTGAAGAACAAGGAACCAACAGAG | GATGGGAAGAGATTAGGGATTGGCTGAT |
| *CmHB15* | CAACGCTAACGAAAGTCCATTGCTACA | GTGACTGATGACGAACAAAGAGAAGAGA |
| *CmHB16* | AAATGGGTGATGGGCAGGAGGGTA | CGTGTAACCGTCCACAGAAGGTAGAA |
| *CmHB17* | CACGACCTCCACGATACCAACCTCTAC | CTGCTGGCTGGTGGTGGGAGTTAA |
| *EF1α* | TTTTGGTATCTGGTCCTGGAG | CCATTCAAGCGACAGACTCA |
